# Three-Dimensional Segmentation and Reconstruction of Neuronal Nuclei in Confocal Microscopic Images

**DOI:** 10.3389/fnana.2019.00081

**Published:** 2019-08-20

**Authors:** Błażej Ruszczycki, Katarzyna Karolina Pels, Agnieszka Walczak, Katarzyna Zamłyńska, Michał Such, Andrzej Antoni Szczepankiewicz, Małgorzata Hanna Hall, Adriana Magalska, Marta Magnowska, Artur Wolny, Grzegorz Bokota, Subhadip Basu, Ayan Pal, Dariusz Plewczynski, Grzegorz Marek Wilczyński

**Affiliations:** ^1^Nencki Institute of Experimental Biology, Polish Academy of Sciences, Warsaw, Poland; ^2^Department of Gene Expression, Institute of Molecular Biology and Biotechnology, Adam Mickiewicz University, Poznan, Poland; ^3^Samsung R&D, Warsaw, Poland; ^4^Center of New Technologies, University of Warsaw, Warsaw, Poland; ^5^Okinawa Institute of Science and Technology, Okinawa, Japan; ^6^Department of Computer Science and Engineering, Jadavpur University, Kolkata, India; ^7^Faculty of Mathematics and Information Science, Warsaw University of Technology, Warsaw, Poland

**Keywords:** chromatin 3D architecture, neurological disorders, epigenetics, neuronal nuclei segmentation, image bioinformatics

## Abstract

The detailed architectural examination of the neuronal nuclei in any brain region, using confocal microscopy, requires quantification of fluorescent signals in three-dimensional stacks of confocal images. An essential prerequisite to any quantification is the segmentation of the nuclei which are typically tightly packed in the tissue, the extreme being the hippocampal dentate gyrus (DG), in which nuclei frequently appear to overlap due to limitations in microscope resolution. Segmentation in DG is a challenging task due to the presence of a significant amount of image artifacts and densely packed nuclei. Accordingly, we established an algorithm based on continuous boundary tracing criterion aiming to reconstruct the nucleus surface and to separate the adjacent nuclei. The presented algorithm neither uses a pre-built nucleus model, nor performs image thresholding, which makes it robust against variations in image intensity and poor contrast. Further, the reconstructed surface is used to study morphology and spatial arrangement of the nuclear interior. The presented method is generally dedicated to segmentation of crowded, overlapping objects in 3D space. In particular, it allows us to study quantitatively the architecture of the neuronal nucleus using confocal-microscopic approach.

## Introduction

The morphological changes in neuronal cell nuclei and the analysis of details of their architecture have recently become an important issue in contemporary neuroscience. Gene expression in the neuronal cell nucleus is known to be crucial for the stabilization and maintenance of synaptic changes underlying the formation of long-term memory (Kandel et al., 2013). The quantitative analysis of the nuclear content has to be preceded by an accurate nuclei reconstruction. Currently, fluorescence confocal microscopy is the leading imaging method for observations of brain samples. Therefore, various computational methods are being constantly developed and improved in order to deal with high-throughput processing of confocal data (Li et al., 2007, 2016; Al-Kofahi et al., 2010; Bilgin et al., 2013; Chen et al., 2013; Kandel et al., 2013; Latorre et al., 2013; Stegmaier et al., 2014; Bajcsy et al., 2015; Mathew et al., 2015; Morales-Navarrete et al., 2015; Hall et al., 2016; Nandy, 2016). A crucial prerequisite to any quantification is a 3D segmentation and reconstruction of neuronal nuclei, which are sometimes tightly packed within the cell layer. Since manual segmentation procedures are too laborious to be practical, one needs an automatic approach. The subject of 2D and 3D automatic segmentation of cellular nuclei has been frequently discussed over the last 30 years and several different approaches have been elaborated (see Stegmaier et al., 2014; Bajcsy et al., 2015), for a short survey. In the case of studies of densely packed neurons, the automatic segmentation of their nuclei is a challenging task. This is mostly due to the fact that the nuclei can lie very close to one another, in such a way that their segmentation is very difficult because of the limited resolution of the light microscopes (especially in the z-direction) and various image artifacts.

A special case is a dentate gyrus (Szczerbal et al., 2009), a part of the hippocampal formation, where the body of granule cells (Hall et al., 2016) are so tightly packed, that the nuclei appear to overlap on microscope images. Thus, we need a method of automatic segmentation and reconstruction of the nuclear content in high resolution confocal stacks, in order to analyze the architecture of neuronal nuclei. There are several biological reasons for performing such an analysis. For example, a range of epigenetic mechanisms has been identified that have important influence on synaptic plasticity (Zovkic et al., 2013). The phenomena that are widely studied in this context include various covalent chromatin modifications (Day and Sweatt, 2011). A few recent studies have pointed to a large scale chromatin remodeling as an additional layer of epigenetic regulation affecting synaptic plasticity (Crepaldi et al., 2013; Walczak et al., 2013; Bharadwaj et al., 2014; Ito et al., 2014). In addition to chromatin, various nuclear inclusions, such as Cajal bodies, PML bodies, and nucleoli were shown to be involved in activity-dependent neuronal plasticity (see Villagra et al., 2008; Hall et al., 2016) and references therein (Hetman and Pietrzak, 2012). All these studies, in a substantial part rely on, or are connected to, the quantitative analysis of neuronal nuclei architecture which is crucial in neuronal differentiation and development (Clowney et al., 2012; Hetman and Pietrzak, 2012; Solovei et al., 2013; Cremer et al., 2015).

The presented method of automatic three-dimensional segmentation and reconstruction of neuronal nuclei was inspired by challenges encountered while analyzing architecture of closely touching neuronal nuclei observed in the hippocampal tissue. This paper presents the methodological details of an algorithm used in already published analyses. However, as the previous studies were oriented toward the understanding of basic biological questions, such as Bdnf gene expression in neuronal nuclei (Walczak et al., 2013), characterization of Histone2GFP mutant mouse nuclei (Ito et al., 2014), or the description of PML nuclear bodies in the brain (Hall et al., 2016), the methodological details of the software were omitted.

The major obstacle in applying the standard methods of segmentation is the fact that each of the nuclei closely touches or overlaps its neighbors in 3D space. While on most of xy-sections they are visibly separated, in 3D they form a bulbous chain difficult to segment by most of the methods. The primary reason for that is lack of the clear border in the overlapping region, the intranuclear inhomogeneities may be more intense than the border that separates the nuclei. Therefore, the generic methods tend to break the nuclei at their inhomogeneities. The second difficulty is the restriction on the size of the analyzed image, as usually the entire data set is loaded into the memory.

The general overview of the presented method is as follows. In confocal microscopy images, densely packed nuclei appear as partially overlying one another in some image planes; such a situation complicates a proper segmentation. However, there are selected image planes, in which such neighboring nuclei are clearly separated. Therefore, we proposed the way to identify such planes, and then to track the nucleus profile, even in the seemingly overlapping regions. Such a tracking allows to assemble the entire three-dimensional nucleus shape.

The effectiveness of the segmentation was manually evaluated for images of different qualities. The correctly recognized segmented nuclei were further processed in order to perform the morphometric measurements of the parameters describing the relations between the internal structures, such as alleles, chromosome territories or nucleoli, and relations between the internal structures and the nucleus boundary. This particular method can be implemented into the program making it capable of dealing with very large data structures because it does not require loading of the whole dataset simultaneously.

## Methods

### Algorithm Overview

The basic idea behind our approach is to reconstruct the nuclear surface of each nucleus starting from the two-dimensional section in a z-plane on which this particular nucleus is well-separated from the adjacent ones. Even for densely packed nuclei, we can find such a section for almost all nuclei. Once we determine the proper boundary of the nucleus on such a two-dimensional section, we can move to the adjacent z-plane. Since the boundary of a nucleus is continuous and does not have any drastic deformations, we know that on the adjacent z-plane, the surface contour differs only slightly from the contour found on the previous section. Due to this fact, we can effectively restrict the region where the nucleus boundary is sought, effectively eliminating the possibility of inclusion of the adjacent nucleus or cutting the segmented nucleus into smaller pieces. The inherent feature of the confocal images is an unequal resolution in the observation plane and toward the optical axis (~3 times better in the x-y directions than in the z-axis). Therefore, a natural choice of the coordinate system is to use the sections perpendicular to the z-axis. An obvious prerequisite for this procedure is to identify for each nucleus, the particular sections in which the nucleus is best separated from its neighboring nuclei. For this purpose, we need to identify a set of seed points lying inside the nuclei which identify the plane at which the boundary detection should start. After the starting z-section is identified, the continuous boundary tracing in the z- positive and negative direction is performed, and all seed points inside the detected nucleus are removed, and the seed point with the highest priority weight from the remaining list is used to initiate the boundary tracing of the next nucleus. This procedure is continued until we go through all seed points.

### Seed Points Detection

**Figure 1:**
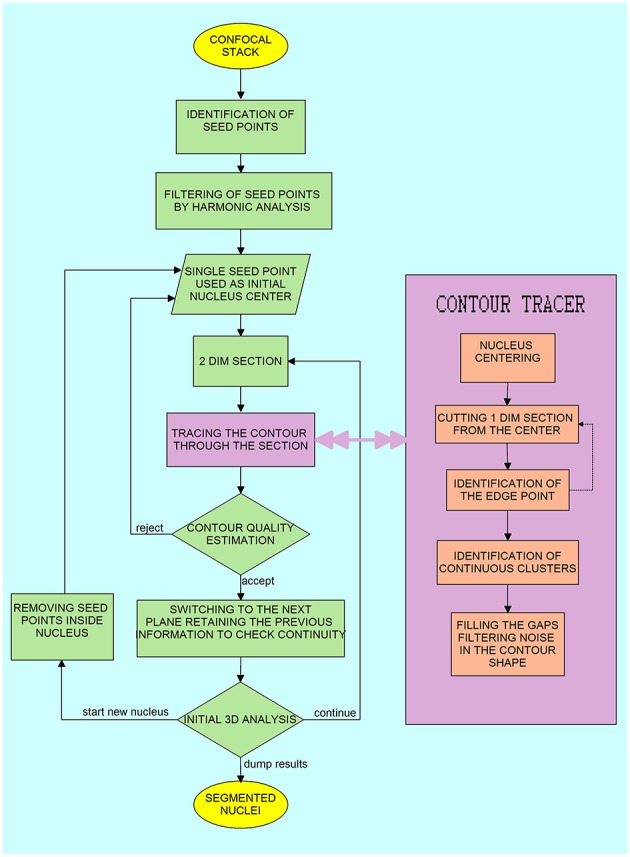
Block diagram of the algorithm for the three-dimensional segmentation and reconstruction of the nucleus surface. The steps in the right inlet “Contour Tracer” operate on a two-dimensional section of the three-dimensional image.

In the preliminary stage of image segmentation, we look for a preliminary set of seed points that defines the tentative centers of mass of nuclei on each 2D section (the first three steps in the algorithm flow, see Figure 1). Therefore, there can be several seed points corresponding to a single 3D nucleus. Some of these tentative seed points are incorrectly assigned (e.g., at the position where two nuclei touch each other), but most of these incorrect seed points will be eliminated in the subsequent procedure. We associate a priority weight (the scalar variable) with each of the seed points. These priority weights are used to sort the set of seed points in the order according to which the procedure of boundary detection is executed. A high priority weight means that the seed point is located at the plane where the nucleus is well-separated from the adjacent ones, and it is therefore advantageous to initiate the segmentation of the corresponding nucleus at this plane. The point with the highest weight does not have to be in the 3D geometrical center of the nucleus. Usually, in the z-dimension, this point is located in the plane where the nucleus is well-separated from the adjacent nuclei, and for this particular z-section, the point is located close to the 2D geometric center of the nucleus in the cutting plane. Such a choice of the seed points greatly facilitated further segmentation of nuclei. Other seed points belonging to the segmented nucleus (with lower priority weights) are removed from the seed point sorted list as we proceed with the segmentation. The position of the seed points is determined by scanning every z-plane independently, and therefore we do not need to load the entire 3D dataset into computer memory.

**Figure 2:**
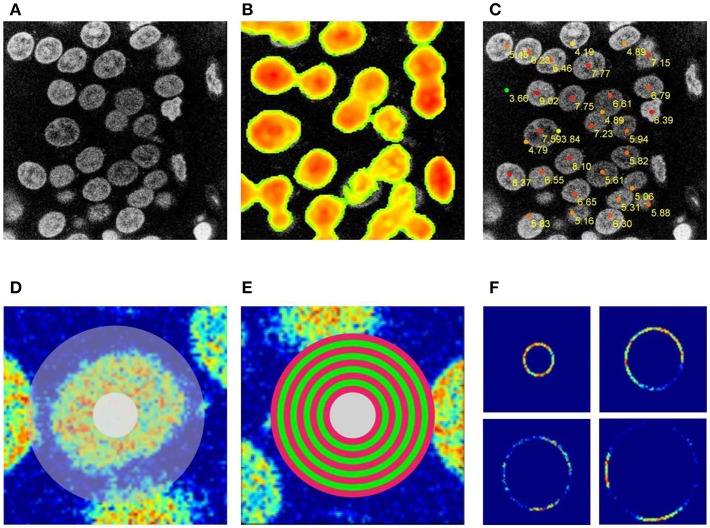
Seed points detection. **(A)** Original image, **(B)** 2D circular filter convolution used to facilitate the detection of neuronal centers on the 2D section–the selection from **(A)**. **(C)** Tentative neuronal centers (red dots) with the priority weights. **(D)** Selection used for procedure of eliminating improper seed points. **(E)** Sequence of rings used to eliminate improper seed points. **(F)** Changes in image brightness for the set of rings shown in **(E)**.

The set of seed points with priority weights is constructed by detecting the local maxima in a convolution of each z-section with circular filters (see Figures 2B,C). The priority weight is the value of the convolution function at the local maximum. Since local maxima often appear at the overlap of two adjacent nuclei, we eliminate these points by applying an iterative method of computing contrast ratios inside rings which cover the approximate pre-defined dimension of the nucleus, entered as initial parameters by the user (see Figures 2D,E), these parameters define the minimal and the maximal diameter of a nucleus on the x-y section, and the maximal and minimal size in the z-dimension (see Supplementary Material SIV for an overview of tunable algorithm parameters). For every ring, we check if the contrast ratio is stable (see Figure 2F) which allows us to eliminate the seed points which are not located around the centers of the actual nuclei. A rapid increase in the fluctuation of the intensity over the circle (for the diameters smaller than the pre-defined minimal nucleus size) was an indication to eliminate the seed point.

Moreover, we compute the shape characteristics of the object calculating the scalar magnitudes of modified version of dipole and quadrupole moments [defined by Equations (1) and (2), respectively] around every potential seed point, which allow us to reject highly irregular shapes which cannot be the sections of a neuronal nuclei,

(1) $$\begin{array}{l} M_{q}=\frac{1}{m}\left(p_{x}^{2}+p_{y}^{2}\right), \end{array}$$

(2) $$\begin{array}{l} M_{d}=\frac{1}{m}det\left(\begin{array}{c} q_{xx}{\;q}_{xy}\\ q_{yx}{\;q}_{yy} \end{array}\right). \end{array}$$

The object whose shape significantly deviates from the oval one has a large value of the dipole moment, and the objects with highly irregular shapes have large values of quadrupole moment, by imposing the limits on the values of these parameters, which were experimentally determined, we can eliminate the seed points that are not associated with the actual nuclei.

To calculate the magnitude of the moments, we need to compute first the modified dipole

(3) $$\begin{array}{l} p_{a}\left(r_{m}\right)=\underset{i}{\sum }s_{a}\left({\vec{x}}_{i},r_{m}\right)\;I\;\left({\vec{x}}_{i}\right)\text{ for }a=x,y, \end{array}$$

and quadrupole moment

(4) $$\begin{array}{l} q_{aa}\left(r_{m}\right)=\sum_{i}s_{aa}\left({\vec{x}}_{i},r_{m}\right)\;I\;\left({\vec{x}}_{i}\right)\text{ for }a=x,y, \end{array}$$

(5) $$\begin{array}{l} q_{xy}\left(r_{m}\right)=\sum_{i}s_{xy}\left({\vec{x}}_{i},r_{m}\right)\;I\;\left({\vec{x}}_{i}\right), \end{array}$$

see (Jackson, 1999).

The quantities appearing in the definitions of dipole and quadrupole moments are defined by Equations (5–8).

(6) $$\begin{array}{l} m=\underset{i}{\sum }\Theta \left(r_{m}-r\right)\;I\;\left({\vec{x}}_{i}\right), \end{array}$$

(7) $$\begin{array}{l} s_{a}\left({\vec{x}}_{i},r_{m}\right)=\Theta \left(r_{m}-r\right)a/{\left(r+\varepsilon \right)}^{1/2},\text{for }a=x,y \end{array}$$

(8) $$\begin{array}{l} s_{aa}\left({\vec{x}}_{i},r_{m}\right)=2\Theta \left(r_{m}-r\right)\left(a^{2}-x^{2}\right)/{\left(r+\varepsilon \right)}^{2},\text{ for }a=x,y \end{array}$$

(9) $$\begin{array}{l} s_{xy}\left({\vec{x}}_{i},r_{m}\right)=2\Theta \left(r_{m}-r\right)xy/{\left(r+\varepsilon \right)}^{2}. \end{array}$$

Here, ${\vec{x}}_{i}$ is the vector pointing to the i-th pixels with coordinates x,y from the chosen origin, $I\left({\vec{x}}_{i}\right)$ is the image intensity at ${\vec{x}}_{i}$ pixel, *r* is the distance between this pixel and the origin, *r*_*m*_ is the radius of the region analyzed, ε is the infinitesimal regularizing parameter assuring the numerical finiteness of the calculated quantities. The step function is defined as

(10) $$\begin{array}{l} \Theta \left(x\right)=1\;if\;x>0;\Theta \left(x\right)=0\;if\;x\leq 0 \end{array}$$

**Figure 3:**
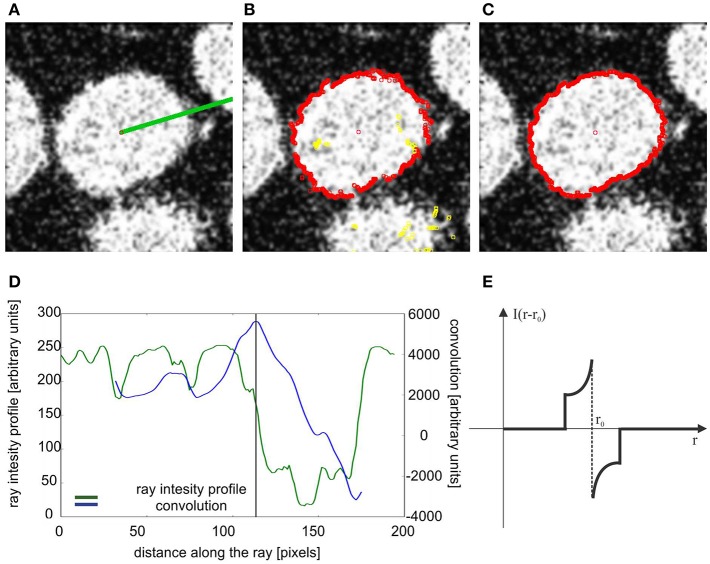
Tracing the nucleus boundary. **(A)** A single ray originating from the seed point. **(B)** Edge points in the first iteration, (yellow edge points were eliminated as laying to far from the clustered points). **(C)** Edge points in the second iteration, **(D)** *green curve*- intensity profile along the ray shown in **(A)**, *blue curve*, convolution of the intensity profile with the convolution mask. **(E)** Convolution mask.

The seed points with the highest weights are therefore located usually in the central regions of nuclei (see Figure 3C).

### Tracing the Nucleus Boundary

The next part of the algorithm performs the tracing of the nucleus boundary at each z-plane. This procedure should deal with nuclei with internal inhomogeneous structure in low contrast conditions with significant variations in image background structure. To perform the tracing we select a seed point and generate a set of one-dimensional rays (Figure 3A, single ray shown) and we extract an intensity profile for each ray (Figure 3D, green curve). For each such ray we calculate a convolution of the intensity profile (Figure 3D, blue curve) with a convolution mask, as in the following equation:

(11) $$\begin{array}{l} F\left(r_{0}\right)=\int drI\left(r\right)M\left(r-r_{0}\right) \end{array}$$

wherein:

- *r* is the distance along the analyzed ray
- *I*(*r*) is the intensity profile on the ray traced from the seed point
- *M*(*r* − *r*_0_) is the mask function (see Figure 3E).

The maximum of the convolution function (11) defines the preliminary position of the boundary points. The collected set of the preliminary boundary points is subject to further processing in order to eliminate points not belonging to the processed nucleus border. To this end, the preliminary boundary points are clustered in the following way: we pick an arbitrary point defining the boundary and move to the adjacent point in a specific direction (e.g., clockwise direction over the boundary). If the distance between this two points is smaller than a specific constant parameter (maximal intracluster deviation, set by the user, see Table S1), the second point is assigned to the same cluster as the first point. We continue the procedure until we find a point that is farther from its neighbor, than the value of the maximal intracluster deviation. In this case we start a new cluster, and continue a procedure. Therefore, we are left with a number of clusters and loose points, that are eliminated. In the next step we choose the cluster with the largest number of points. We move to the ray that defined the point, that was adjacent to this cluster, but could not be assigned into the cluster. For this ray we look for the new position of the boundary point, at this time restricting it to the maximal distance defined by the value of maximal intracluster deviation parameter. This new point will necessary belong to the adjacent cluster. This procedure is continued until we trace the entire 2D boundary. Figure 3B shows all points during the first tracing- the points marked by yellow were eliminated during the described procedure. Figure 3C shows all points after reiterating, starting from the cluster with the largest number of points.

At the beginning of segmenting a specific nucleus, we analyze only a single z-plane and perform two iterations of boundary tracing. The first iteration roughly localizes the boundary in a neighborhood to which the second iteration is restricted and produces a contour. In the next step, we consider the adjacent z-plane and restrict to a tubular neighborhood of the projection of the contour found in the previous step and produce a contour for the new z-plane. We continue in this way until a z-plane is reached where the produced contour is approximately a point. The seed points which lie within calculated boundary are then eliminated from the seed point set.

For each traced 2D contour, we calculate the quality estimator Q, based on the ratio of the integrated intensity in the laminar layers inside and outside the nuclear surface according to the following procedure:

First, for each point on the boundary we calculate the integrated image brightness on the laminar layer on both side of boundary, *L*_+_ and *L*_−_ as

(12) $$\begin{array}{l} L_{-}\left(r_{0}\right)=\overset{r_{0}}{\underset{r_{0}-\Delta r}{\int }}drI\left(r\right), \end{array}$$

(13) $$\begin{array}{l} L_{+}\left(r_{0}\right)=\overset{r_{0}+\Delta r}{\underset{r_{0}}{\int }}drI\left(r\right), \end{array}$$

where is the parametrization of the contour by the ray from the mass center of the contour, *I* is image brightness in the center point, Δ*r* is the width of the laminar ray.

Next, we calculate the *Q* as

(14) $$\begin{array}{l} Q=\langle \Theta \left(L_{+}/L_{-}\right)\rangle \end{array}$$

where the mean is taken over all boundary points for given section. If this value is above the user set threshold e.g., we proceed with the boundary tracing on the next z-section and continue the segmentation by analyzing the neighboring sections, otherwise the procedure is discontinued and the respective seed point is abandoned. The procedure is also terminated when we reach the end nucleus, this end is determined based on the fact that there is too little fluorescence intensity in the analyzed region, or the area of the traced contour starts to grow larger after the monotonic decrease.

## Materials

### Tissue Preparation

All experiments performed on animals were carried out in accordance with relevant guidelines and regulations and were approved by the First Warsaw Local Ethics Committee for Animal Experimentation, approval number 1015/2009. Rats and mice were lethally anesthetized with sodium pentobarbital (Biowet Pulawy) in a dose of 100 mg/kg body weight, diluted in saline, and perfused immediately with 0.1 M phosphate-buffered saline (PBS) (pH 7.4), followed by cold 4% paraformaldehyde (Sigma Aldrich, cat. No. P6148), in PBS (pH 7.4). The brains were cautiously removed from the skulls and placed for 24 h in the same fixative at 4°C. Then the brains were cryoprotected with 30% solution of sucrose in PBS, frozen in the −80°C cold n-heptane, and stored at −80°C. until needed. Forty-micrometer-thick free-floating sections were cut coronally at −20°C with the use of the cryostat and stored in anti-freeze solution (30% glycerol; 30% ethylene glycol; 0.03 M NaH_2_PO_4_; 0.01 M NaOH; distilled water), preventing the formation of freezing artifacts. The sections were subjected to DNA in situ hybridization and/or immunofluorescence, as described in Ito et al. (2014) and Hall et al. (2016), respectively.

### Image Acquisition

To exercise the algorithm we used confocal image-stacks visualized exclusively for the purpose of this manuscript and confocal images used as controls in our three former publications (Walczak et al., 2013; Ito et al., 2014; Hall et al., 2016). In total, we used 36 confocal stacks, imaging brain tissue of 19 rodents (16 rats and three mice), we collected up to three stacks per animal. The maximal imaging depth in z-direction was 170 planes, and up to 2,048 × 2,048 pixels in x-y direction.

Granular layer of the dentate gyrus was examined under the spectral confocal microscope TCS SP5 (Leica), using 488 nm Ar, 561 nm DPSS diode, and 633 nm HeNe laser lines for the excitation of FITC/Alexa488/Dylight488/Qdot525, Rhodamine/Alexa546, and Cy5/TOPRO-3, respectively. To image Hoechst33342, a two photon excitation with Mai Tai IR femto operating at 720 nm was used. The images were acquired through the internal TCS SP5 detectors/photomultipliers. To avoid cross talk between the fluorophores, we carefully adjusted the spectral ranges of the detectors and scanned images sequentially. The planar apochromatic oil-immersion objective lenses were 20 × (0.7 NA), and 63 × (1.4 NA). The image stacks of FISH/immuno-FISH were acquired at the lateral resolution of 80 nm/pixel, with Z-spacing of 200 nm. To reduce noise and improve resolution, the stacks were 3D deconvolved by means of Huygens Professional software (Scientific Volume Imaging), using the classical maximum likelihood algorithm and theoretical point-spread functions. During imaging of mouse neuronal nuclei, the exposition was increased to oversaturate partially the images (the bright chromocenters in the nuclei) what resulted in more homogeneous nucleus texture. In the case of observation of nuclei with aberrant chromatin pattern the nucleus boundary was enhanced using anti-lamin B immunostaining (Ito et al., 2014). For final inspection and publication, brightness and contrast of the images were manually adjusted.

### Method Availability

The source files (Python scripts) and the exemplary numerical data used for analysis are available from https://gitlab.com/pnmis/nuclear-segmentation.git.

## Results

### Algorithm Performance

We performed segmentation and analysis of several confocal stacks of rat and mouse brain tissue (see Materials, Image Acquisition, for the imaging details and the number of subjects), including samples from mutant mice with highly aberrant structure of neuronal nuclei (Ito et al., 2014). The major steps in the presented approach of nuclei segmentation and reconstruction are: (a) seed point detection, (b) 2D contour tracing, (c) reconstruction of the nuclear boundary, and (d) reconstruction of internal objects, (see section Methods and the block diagram of the segmentation algorithm presented in Figure 1). From the module of seed point detection we obtain several seed points per single nucleus (see Figure 2), the point with highest priority weight (see Methods–seed point detection for details) is used to begin the segmentation of the nucleus boundary around it (see Methods–tracing nucleus boundary, and Figure 3 for details). The procedure of contour tracing and reconstruction of the entire 3D nuclear boundary interact with each other, these procedures are executed until all the seed points are used.

At every stage of the segmentation method we encounter specific artifacts that have to be taken into account. These artifacts include a ubiquitous noise in images and inhomogeneities of the nuclear structure. The space between nuclei is often filled with particles of various origins, usually due to unspecific binding of DNA stain. These artifacts often interfere with the boundary of the nuclei. In addition, the internal structure of the nuclei introduces a significant inhomogeneity in the chromatin texture, such as chromocenters (especially in mouse species). Altogether, the aforementioned problems prevented the use of standard methods and various morphological filters (see Supplementary Material SI) to perform the segmentation.

**Figure 4:**
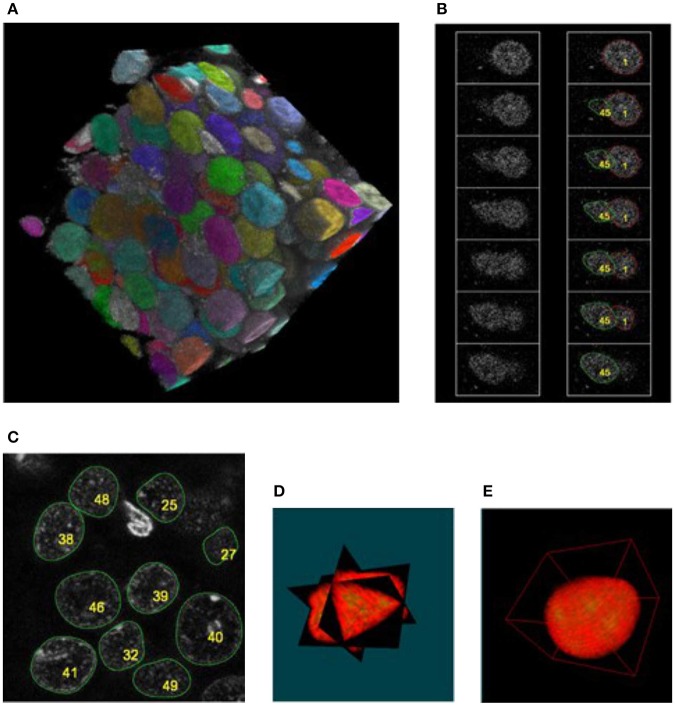
Segmentation of the confocal stack showing fluorescently labeled hippocampal neuronal nuclei in the rat brain; **(A)** the segmented nuclei were randomly colorized for illustrative purposes, stack No. 1; **(B)** adjacent z-sections of the closely touching nuclei where the segmentation was ambiguous, nevertheless the method was able to separate the overlapping structures, stack No. 1; **(C)** segmentation of the nuclei with inhomogeneous internal structure, stack No. 4; **(D,E)** orthogonal views and 3D rendering of the single nucleus extracted from the confocal stack. See Supplementary Material SIII, for more details.

The presented method was tested by applying it to segment seven different confocal stacks containing cellular nuclei (see Table 1 for their detailed description). Figure 4A shows the completed segmentation of 1,024 × 1,024 × 161 confocal stack (stack No. 1), where the nuclei were randomly colorized while we kept the original image intact in the inter-nuclei space. The detail revision reveals a presence of nuclei which overlap in the confocal image so closely that it is ambiguous to decide where is the actual boundary between them (Figure 4B), moreover it appears that there is a “common part” where the images of both nuclei are superposed. Only the nuclei fully contained in the confocal stack (not cut by the boundary walls) were considered (Figure 4C), the cropped nuclei had to be rejected as not appropriate for the most of morphometric measurements. Quite often the contour reconstruction on the first or last z-plane containing nucleus was not precise resulting from the poor image quality at these planes, due to confocal microscope image modalities. In Figures 4D,E rendering of a single nucleus with additional channels displaying other fluorescent signals is presented.

**Table 1:** Validation of the segmentation results.

**Stack no**.	**Stack description**	**Origin**	**PS**	**NP**	**OS**	**US**	**FP**	**ND**
1.	High contrast, nuclei uniformly labeled Size: 1,024 × 1,024 × 161 px 71.68 × 71.68 × 33.81 μm	Rat, dentate gyrus of the hippocampus	97	48	8	1	18	7
2.	High contrast, nuclei with many small internal spots size: 1,024 × 1,024 × 96 px 71.68 × 71.68 × 20.16 μm	Rat, Dentate gyrus of the hippocampus	61	31	10	0	6	4
3.	High contrast, nuclei with large internal spots Size: 1,024 × 1,024 × 138 px 71.68 × 71.68 × 28.98 μm	Mouse, Somatosensory cortex	47	21	5	1	11	2
4.	Low contrast, nuclei non-uniformly labeled Size: 1,024 × 1,024 × 112 px 71.68x71.68x23.52 μm	Mouse, Somatosensory cortex	57	34	3	4	6	3
5.	Low contrast, nuclei uniformly labeled Size: 1,024 × 1,024 × 164 px 71.68 × 71.68 × 34.44 μm	Mouse, Somatosensory cortex	59	35	14	6	9	4
6.	Low contrast, nuclei with internal spots Size: 1,024 × 1,024 × 176 px 71.68 × 71.68 × 36.96 μm	Rat, Dentate gyrus of the hippocampus	36	20	23	1	2	12
7.	Low contrast, nuclei with many internal spots Size:1,024 × 1,024 × 38 px 71.68 × 71.68 × 7.98 μm	Rat, Dentate gyrus of the hippocampus	13	27	1	0	2	59

*The following definitions have been used: PS, precisely segmented; NP, not-precisely segmented; OS, over segmented; US, under segmented; FP, false-positive; and ND, not detected. Examples of slices from each stack are presented in Figure S5 of Supplementary Materials*.

Table 1 presents the quantitative estimates of the accuracy of our method. During manual verification we count separately the cases of precise segmentation (PS)—when during the visual inspection we did not observe defects in the reconstruction of the nuclear volume, and the cases of non-precise segmentation (NP)–when we observed defects in reconstruction of the nuclear volume (up to 5% of voxels), or the nuclei were seemingly overlapping due to the poor confocal resolution in the z-direction. As the perfectly reconstructed nuclear surface is crucial for quantitative studies, we selected only the nuclei belonging to the first category, PS. The nuclei belonging to the NP category may still provide valuable information for some experimental questions. The other rejected nuclei belong to the following categories: over segmented (OS)–denoting the case where one nucleus has been segmented as multiple ones; under segmented (US)–denoting the case where two (or more) nuclei have been classified as a single one; false-positive (FP)–being the detections not corresponding to any nuclei; not detected (ND)–when the entire nucleus has been missed. For the vast majority of confocal stacks we obtain satisfactory numbers of PS nuclei varying between 46 and 54% of the total. Due to a very low signal/noise ratio, for the confocal stack No. 7 we have not obtained satisfactory results of segmentation. The quality of these images was so poor that even visual inspection of the nuclei did not allow for full unambiguous recognition of the individual nuclei (see Supplementary Material SII).

In Table 2 we present the values of quantitative measures describing quality of segmentation results. We are using the following measures: recall, precision, F-measure, and accuracy, which are given by the subsequent equations:

(15) $$\begin{array}{l} \text{recall}=\text{TP}/\left(\text{TP }+\text{ FN}\right), \end{array}$$

(16) $$\begin{array}{l} \text{precision}=\text{TP}/\left(\text{TP }+\text{ FP}\right), \end{array}$$

(17) $$\begin{array}{l} \text{F-measure}=2^{*}\text{recal}{\text{l}}^{*}\text{precision}/\left(\text{recall }+\text{ precision}\right), \end{array}$$

(18) $$\begin{array}{l} \text{accuracy}=\text{TP}/\left(\text{TP}+\text{FP}+\text{FN}\right) \end{array}$$

where TP, true positive, FP, false positive, FN, false negative (see Mathew et al., 2015).

**Table 2:** Quantitative analysis of the segmentation results (quality II/quality I).

**Stack no**.	**TP**	**FP**	**FN**	**Recall**	**Precision**	**F-measure**	**Accuracy**
1.	145/97	27/75	7/7	0.95/0.93	0.84/0.56	0.9/0.7	0.81/0.54
2.	92/61	16/47	4/4	0.96/0.94	0.85/0.56	0.9/0.71	0.82/0.54
3	68/47	17/38	2/2	0.97/0.96	0.8/0.55	0.88/0.7	0.78/0.54
4	91/57	13/47	3/3	0.97/0.95	0.88/0.55	0.92/0.7	0.85/0.53
5	94/59	29/64	4/4	0.96/0.94	0.76/0.48	0.85/0.63	0.74/0.46
6	56/36	26/46	12/12	0.82/0.75	0.68/0.44	0.75/0.55	0.6/0.38
7	40/13	3/30	59/59	0.40/0.18	0.93/0.3	0.56/0.23	0.39/0.13

TP, true positive (positively segmented); FP, false positive (segmented with defects: NP, OS, US, and FP); FN, false negative (not detected). recall = TP/(TP + FN), precision = TP/(TP + FP), F-measure = 2^*^recall^*^precision/(recall + precision), accuracy = TP/(TP+FP+FN) (see Mathew et al., 2015). Quality I: reconstruction of nucleus surface without defects, quality II: defects, up to 5% of voxels incorrectly assigned.

This analysis does not take into account nuclei segmented with defects. We therefore introduce additional categories shown in Table 1. We include all nuclei segmented with some defects (i.e., NP, OS, and US) to the FP category. The TP nuclei are those which were correctly segmented (PS), the false negative are those which were not segmented at all (ND).

**Figure 5:**
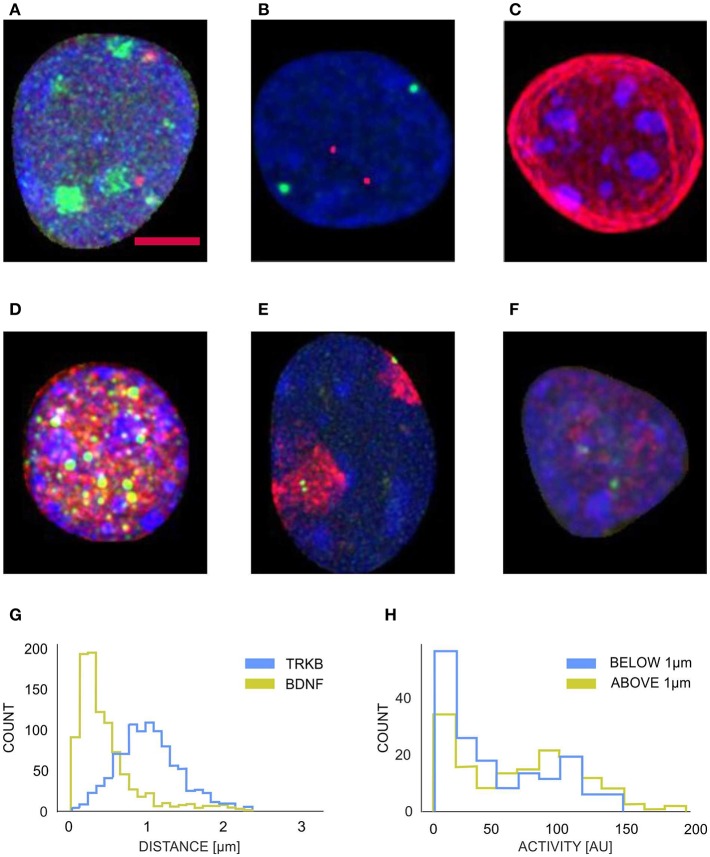
Examples of segmented neuronal nuclei with different intraneuronal structures labeled fluorescently (z-projections) **(A–F)** and quantification **(G,H)**: **(A)** blue, DNA; red, BDNF alleles, green-nucleoli; **(B)** blue, DNA; red, TRKB alleles; green, BDNF alleles; **(C)** blue, DNA (ungrouped chromocenters); red, Lamin; **(D)** blue, DNA grouped chromocenters); red, NeuN; green, PML bodies; **(E)** blue, DNA; red, Chromosome 3 territory; green, BDNF alleles; **(F)** blue, DNA; red, Polimerase activity; green, BDNF alleles. The labelings of the interneuronal structures were used to perform a series of morphometric measurements. **(G)** Represenative graph of distributions of the distances (*d*) of *Bdnf* (green histogram) and *Trkb* alleles (blue histogram) from the nuclear periphery. *Bdnf* : *d* = 0.499 ± 0.034 μm, *Trkb*: *d* = 1.069 ± 0.036 μm, *p*-value (Mann–Whitney test) < 0.001, *p*-value (Kolmogorov-Smirnov test) < 0.001, *N* = 12, *n* = 963 (animals, nuclei; respectively). **(H)** Graph presenting distribution of the intensity of RNA polymerase II immunoreactivity at the *Bdnf* alleles located more or <1 μm from the nuclear periphery (green and blue histogram, respectively). Activity (peripheral alleles): 47.77 ± 1.75 [a.u.], activity (internal alleles): 65.43 ± 2.24 [a.u.], *p*-value (Mann-Whitney test) = 0.006, *p*-value (Kolmogorov–Smirnov test) < 0.001, *N* = 5, *n* = 310 (animals, nuclei; respectively). Scale bar: 2 μm.

After parameterizing nuclear membrane, we proceeded toward the reconstruction of objects contained in the interior of the nucleus and perform a series of morphometric measurements. We evaluated various signals by means of fluorescent *in situ* hybridization (FISH) and/or immunofluorescence including imaging of genes, chromo-some territories, nucleolus, and RNA polymerase transcription factories (see Figures 5A–F). These measurements can combine the spatial arrangement of the observed objects with intensity information from multi-channel images. The parameterization of the nuclear membrane was used to calculate the nucleus volume, the membrane area, the shape form factor, and in conjecture with the allele coordinates, to calculate the relative positions of the alleles (e.g., the distances between the alleles and the nucleus envelope), these parameters play a significant role in analysis of gene movements (Kosak et al., 2002; Zink et al., 2004; Ragoczy et al., 2006; Williams et al., 2006; Szczerbal et al., 2009; Peric-Hupkes et al., 2010; Clowney et al., 2012; Solovei et al., 2013). The coordinates of the alleles were obtained by segmenting them with Otsu thresholding, the unspecific punctuate signal of lower brightness originating from unspecific binding from the probes to the nuclear proteins or statistical fluctuations of the detector noise was eliminated by calculating integrated brightness for each object and selecting only two objects with the largest integral value. Figure 5G shows that TRKB allele is position significantly further from the nuclear envelope, than the BDNF allele, which is located in the close proximity (mostly <0.5 μm) of the nuclear lamina. Figure 5H shows the histogram of the transcriptional RNA polimerase factory for different locations of BDNF allele. We observe a larger proportion of alleles with smaller polimerase activity, for the selection of alleles located in the proximity of nuclear lamina (<1 μm). Such analysis can also reveal a special relationship between different genes (Kosak et al., 2002; Zink et al., 2004; Ragoczy et al., 2006; Williams et al., 2006; Szczerbal et al., 2009; Peric-Hupkes et al., 2010; Clowney et al., 2012; Solovei et al., 2013), this topic has been recently intensively studied in non-neuronal cells, as spatial arrangement is constantly more and more recognized as an import relation influencing gene expression.

### Comparison With Other Methods

**Figure 6:**
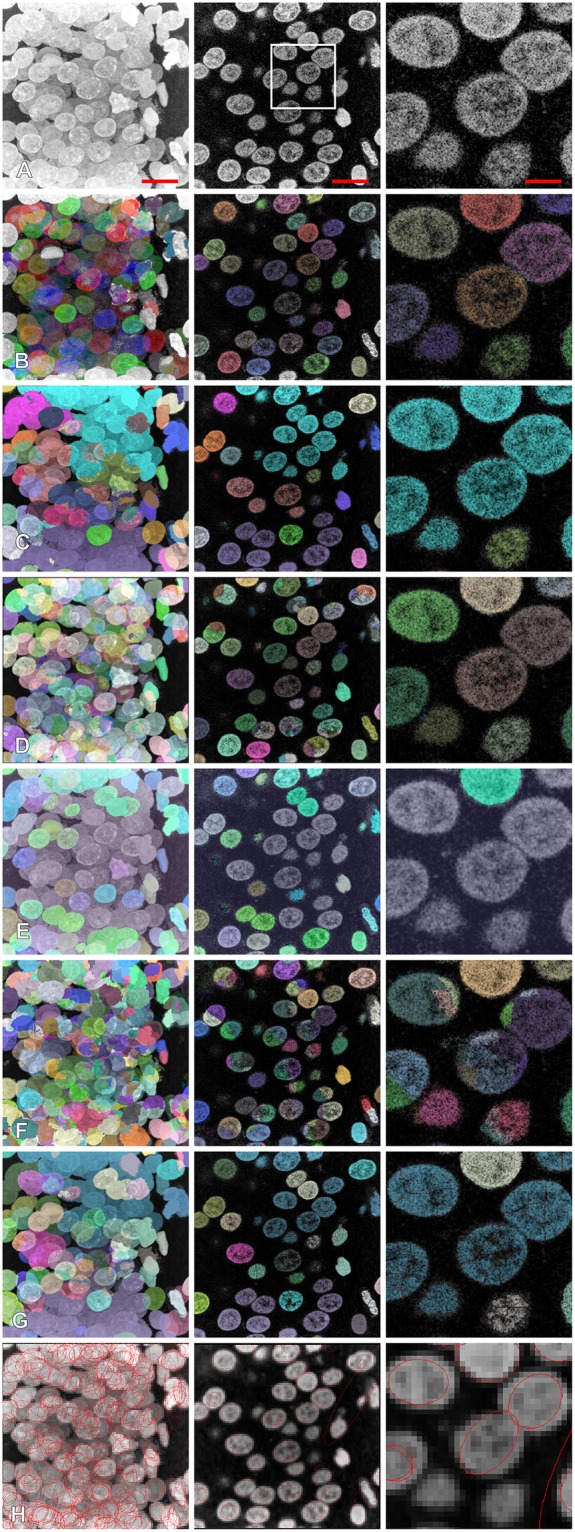
Comparison of segmentation results with different other methods: **(A)** original image, **(B)** proposed method, **(C)** Ilastik, **(D)** gradient flow tracking, **(E)** MorphoLibJ, **(F)** Farsight, **(G)** classical watershed, **(H)** method based on the curvatures of the iso-intensity surfaces. Left column: z-projection, middle column: z-section, right column: magnification. Scale bars: 7 μm—left, middle, 2.3 μm—right. Segmented nuclei were randomly colorized for illustrative purposes **(A–G)**, for **(H)** contours of ellipsoids representing the segmented results are shown.

We compared the results of segmentation of the proposed method with six other available methods Ilastik (Sommer, 2011), gradient flow (Li et al., 2007), MorphoLibJ (Legland et al., 2016), Farsight (Narayanaswamy et al., 2010), classical watershed (Vincent and Soille, 1991) curvatures of the iso-intensity surfaces (Toyoshima et al., 2016) (see Table 3 and Figure 6). For the methods (Li et al., 2008; Narayanaswamy et al., 2010; Legland et al., 2016; Toyoshima et al., 2016), we tried several different sets of parameters, and chose the set giving the best performance, the Ilastik method described in Sommer (2011) required an initial training on a sample data set. For the methods (Li et al., 2008; Toyoshima et al., 2016), we had to downsample the image resolution (respectively, by a factor 2 and 4) as the hardware memory (16GB) did not suffice to perform the segmentation. For both classical watershed (Vincent and Soille, 1991) and gradient flow (Li et al., 2008) methods, we observed the presence of few blobs containing several undersegmented nuclei. The segmentation performed by MorphoLibJ (Legland et al., 2016) resulted in a significant portion of undetected nuclei. The methods (Li et al., 2008; Narayanaswamy et al., 2010; Toyoshima et al., 2016) led to a large fraction of oversegmented nuclei. For the method based on analysis of curvatures of the iso-intensity surfaces (Toyoshima et al., 2016), we obtained a high proportion of nuclei correctly segmented (yet “not precise” according to our classification), this deviation was mainly due to the fact, that the method (Toyoshima et al., 2016) assumes the ellipsoidal shape of the nuclei (shown as contours in Figure 6G), which deviate from the actual shapes of the nuclei in our sample. The number of segmented nuclei in Category I by the proposed method outperformed each comparison example.

**Table 3:** Comparison of the segmentation results, for different methods.

	**PS**	**NP**	**OS**	**US**	**FP**	**ND**	**Recall**	**Precision**	**F-measure**	**Accuracy**
Proposed	97	48	8	1	18	7	0.95/0.93	0.89/0.84	0.92/0.89	0.85/0.79
Ilastik	15	11	3	9	0	2	0.93/0.88	1.0/1.0	0.96/0.94	0.93/0.88
Gradient flow	34	26	43	13	15	0	1.0/1.0	0.8/0.69	0.89/0.82	0.8/0.69
MorphoLibJ	18	5	0	12	4	122	0.16/0.13	0.8./0.7	0.27/0.22	0.15/0.12
Farsight	6	5	76	4	22	55	0.16/0.1	0.33/0.21	0.22/0.13	0.12/0.07
Classical watershed	25	8	5	9	0	6	0.85/0.8	1.0/1.0	0.86/0.6	0.84/0.8
Curvatures of the iso-intensity surfaces	23	69	174	35	30	0	1.0/1.0	0.75/0.43	0.92/0.85	0.75/0.43

*See captions at Tables 1, 2 for explanations of notation*.

In order to perform the cross-check of morphometric measurements (nuclear volume and surface, the distances between the surface of the alleles and the nuclear boundary, the distances between the surfaces of the alleles) we created artificial ellipsoidal nuclei with internal objects with a'priori known dimensions, position and arrangement. The artificial nuclei were created in 3D Studio Max software, as ellipsoids with assumed arrangement and minor and major axis. Inside the ellipsoids we placed two small spheres on each channel, which mitigated the alleles. Subsequently, we added the Gaussian noise. For these artificial nuclei we calculated analytically the aforementioned morphometric parameters. These values were in a good agreement (discrepancy resulting from numerical accuracy) with the values obtained numerically from the image analysis.

## Discussion

An automated segmentation and surface reconstruction of neuronal nuclei is a crucial procedure required for quantitative studies of neuronal architecture. Crowding of nuclei and their varying structure (presence of chromocenters, inhomogeneities, and overlapping nuclei) are major obstacles for automatic segmentation. The presented method, which relies on very mild assumptions on the nuclear shapes, is capable of resolving a large number of nuclei allowing further quantitative analysis. Nevertheless, the results of the segmentation still required manual verification in order to reject the improperly segmented cases.

The majority of the nuclei for the confocal stacks we further processed were correctly segmented, yet we encountered a significant number of nuclei which contained defects (with no under- or over segmentation) in their surface reconstruction (see Tables 1, 2). These were mostly overlapping nuclei with no border between them, for which the segmentation method is not capable to properly determine the border between the nuclei (any determined boarder is based on a sort of extrapolation), such nuclei were classified into NP category (non-precisely segmented). Moreover, we accounted the glial cells into the FP (false positive) category, we did not include into the algorithm the criteria whether a nucleus is from the glial cell or not. Still, we are able to extract a sufficient number of nuclei to reveal several morphological features, e.g., differences in location of BDNF and TRKB alleles, see Figure 5G. The presented method allowed for the analysis of more than 4,000 nuclei, segmented from the hippocampal dentate gyrus (Walczak et al., 2013).

The hippocampal dentate gyrus, in our experience, is the most extreme case of nuclei crowding. We expect, that nuclei in other brain areas, are easier to segment, we do not however foresee the major differences between the rodents and other species. The crucial factor influencing the image modality seems to be the brain region.

We were able to segment confocal stacks of dimensions up to 1,024 × 1,024 × 176 voxels in computational time of <2 h using a single processor core. The speed of the method could be increased via parallel processing since the seed point set can be divided into subsets corresponding to different nuclei and independently processed. However, parallelization in Python (in which we developed the algorithm) is a complex task, due to the presence of the GIL (Global Interpreter Lock). Therefore, we decided to run, in parallel, segmentation of different stacks, each as a separate process. As some of the confocal stack are pretty large (e.g., 4,096 × 4,096 × 300 voxels), the segmentation of multiple images at the same time is restricted by the computer memory. However, the advantage of the proposed method is that it does not require loading the full data set into memory, more specifically, it only needs simultaneous loading of two slices, at the cost of re-reading the data which consumes up to 30% of total segmentation time. If segment smaller stacks, the computer memory is usually sufficient to load all data for the bunch of used processes. With the larger stacks it pays off to load only the required part of the data, allowing to segment more stacks simultaneously.

Another improvement of the algorithm could be to develop a post-processing procedure to correct the quality of the surface reconstruction of the nuclei that were classified as non-precisely segmented. The difficulty in developing a post-processing procedure is the variety of artifacts that influence the surface quality.

The presented method requires to adjust manually few parameters with varying data modalities. The main parameter values that need to be set manually are: maximal and minimal nucleus size (separately for xy- and z-dimension), minimal value of priority weight associated with a seed point (this value is set experimentally by analysis of a single section, setting too larger value results in omission of nuclei, setting too small values results in abnormal computational time as the algorithm tries to find nuclei in the background noise), the parameter controlling how much the boundary varies between two adjacent plane (setting too large value results in under segmentation, too small value results in omission of nuclei). The other parameters control the numerical accuracy, e.g., the resolution of points parameterizing the boundary, or the minimal value of the quality estimator Q, that controls when to abort the segmentation of nucleus, when the procedure fails. In summary, an appropriate adjustment is necessary to compensate the variability in image quality.

Even if performing manual segmentation, one often extrapolates the nucleus surface at the locations where the artifacts or irregularities occur (e.g., holes or overlapping nuclei). To recognize automatically such irregularities we need to construct a model of the nucleus by which we can decide whether a certain image feature is an artifact or not. Any such model, however, would be strongly determined by the details of the features of analyzed nuclei (e.g., it would be strongly influenced by an aberrant nuclear structure), and therefore it would suffer a loss of generality. The model based recognition may lead to the bias resulting from the selective recognition of objects which fit to the a-priori implemented model (Wienert et al., 2012). Thus, the presented algorithm was based on very general assumptions concerning the neuronal shape, rather than on the specific nucleus model, asserting that (a) two adjacent z-sections of the nuclei, which are not very thick (210 nm), do not vary dramatically from each other (the condition of the continuity of the nuclear membrane), (b) for each nucleus, there exists a section where the nucleus is well-separated from the adjacent nuclei, since even densely packed nuclei cannot fill the whole space, and (c) the shape of the nuclei is convex. We did not assume the homogeneity of the fluorescence from the marker used to stain the nuclei, allowing for analysis of nuclei with disrupted architecture (Ito et al., 2014). These assumptions are however challenged by various image artifacts, whose presence limits the effectiveness of the proposed image processing method.

## Data Availability

The datasets generated for this study are available on request to the corresponding author.

## Author Contributions

BR invented the method. BR and GW directed the research and wrote the manuscript. BR, SB, DP, and AP developed the software. GB and DP contributed by preparing Python bindings and performing tests of the software. MS, KP, and KZ optimized, tested, and validated the software. AS, AM, MH, MM, and AWa prepared the biological samples and collected the images. DP provided critical comments on the method and the manuscript. AWo contributed by preparing artificial model to test the software.

### Conflict of Interest Statement

BR and GW are owners of the Patent EP2549433A1. We declare that there are no other competing financial and non-financial interests. KZ is employed by Samsung R&D, Warsaw, Poland. The remaining authors declare that the research was conducted in the absence of any commercial or financial relationships that could be construed as a potential conflict of interest.
