# Supplementary material for: Three-Dimensional Segmentation and Reconstruction of Neuronal Nuclei in Confocal Microscopic Images
Source: Front Neuroanat. 2019 Aug 20;13:81. doi: 10.3389/fnana.2019.00081 (PMC6710455; doi:10.3389/fnana.2019.00081)
Supplement: Supplementary file 1 [file Data_Sheet_1.PDF]

SUPPLEMENTARY MATERIALS

Three-Dimensional Segmentation and Reconstruction of Neuronal Nuclei in Confocal Microscopic Images.

Błażej Ruszczycki*[1], Katarzyna Karolina Pels[1], Agnieszka Walczak [2], Katarzyna Zamłyńska[3], Michał Such[1], Andrzej Antoni Szczepankiewicz[1], Małgorzata Hanna Hall[1], Adriana Magalska[1], Marta Magnowska[1], Artur Wolny[1], Grzegorz Bokota[4], Subhadip Basu[5], Ayan Pal[5], Dariusz Plewczynski[4,6], Grzegorz Marek Wilczyński*[1]

[1] Nencki Institute of Experimental Biology, Polish Academy of Sciences, Warsaw, Poland, [2]Department of Gene Expression, Institute of Molecular Biology and Biotechnology, Adam Mickiewicz University, Poznan, Poland [3]Samsung R&D, Warsaw, Poland, [4]Center of New Technologies, University of Warsaw, Poland, [5] Department of Computer Science and Engineering, Jadavpur University, India, [6]Faculty of Mathematics and Information Science, Warsaw University of Technology, Warsaw, Poland.

S I. Effects of preprocessing of the image with morphological filters

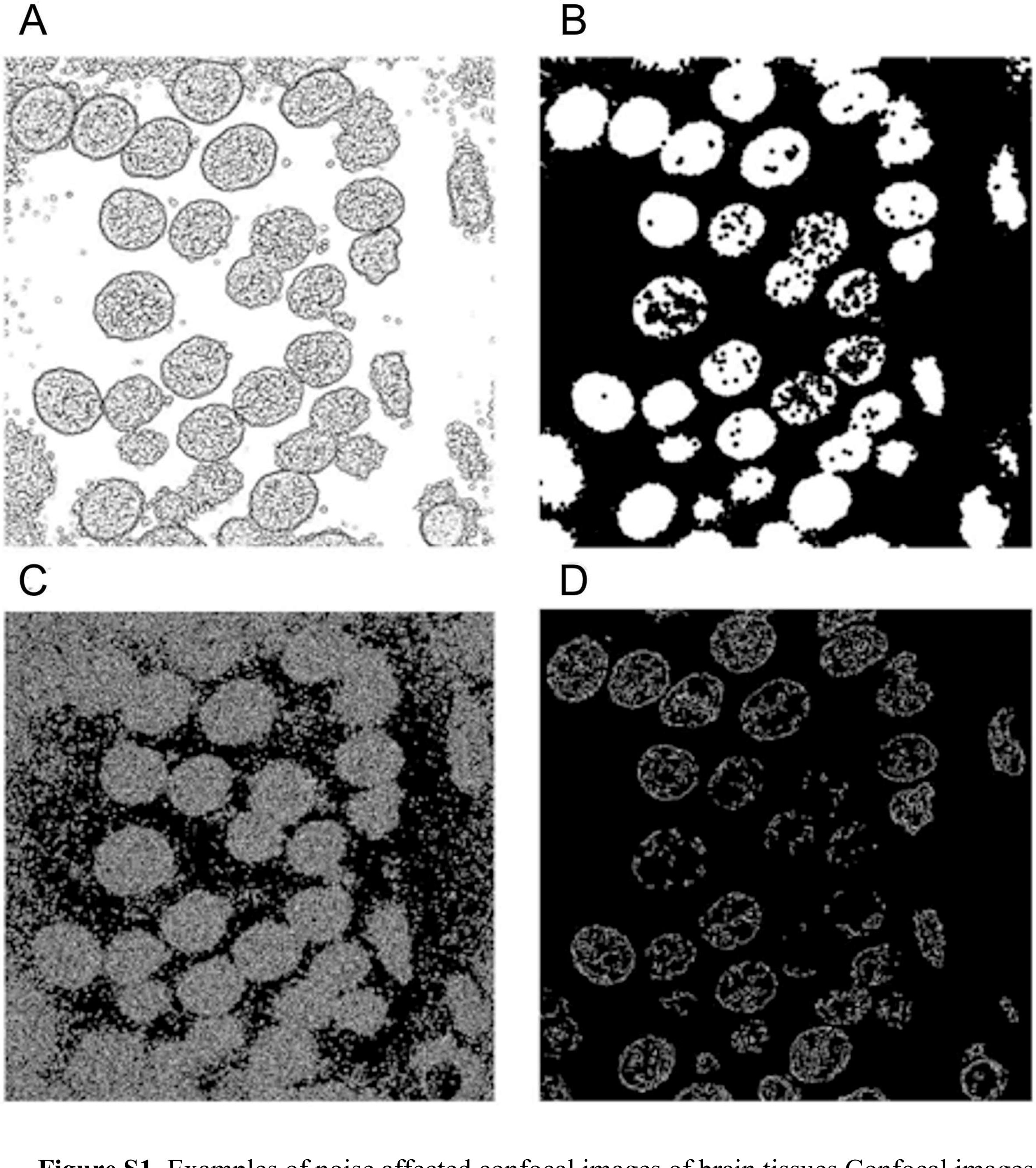

Figure S1. Examples of noise affected confocal images of brain tissues.Confocal images of brain tissue are heavily affected by noise. Limited light environment makes images prone to diverse artifacts. Noise appears both on the inside of nuclei where it disrupts its homogeneity and on the outside making nuclei difficult to separate. Those artifacts are the major obstacle to use simple morphological filters (i.e. erosion or dilation) or more sophisticated ones such as watershed. It may be possible to find set of filters for a given image but it requires human interaction and due to the fact that every study would need different filter set, this approach is not feasible to automate filtering.

S II. The dependence of segmentation accuracy on the quality of initial data

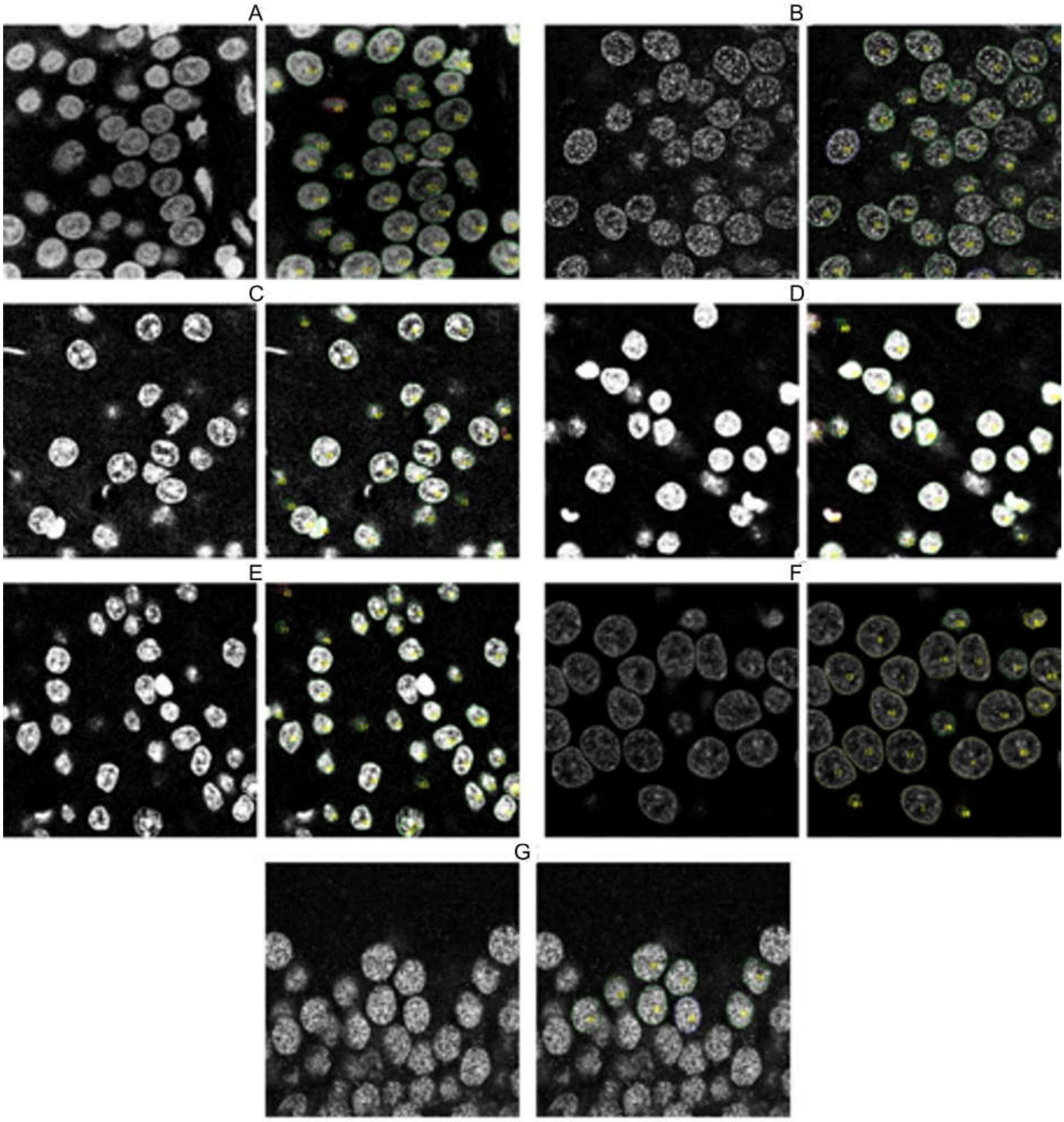

Figure S2. Initial data and the segmentation for different data sets (single slices) using propose method, see Table 1 for details. (A) stack No. 1, z-section No. 40. (B) stack No. 2, z-section No. 40. (C) stack No. 3, z-section No. 52. (D) stack No. 4, z-section No. 76. (E) stack No. 5, z-section No. 76. (F) stack No. 6, z-section No. 30. (G) stack No. 7, z-section No. 13.

S III. Additional figures

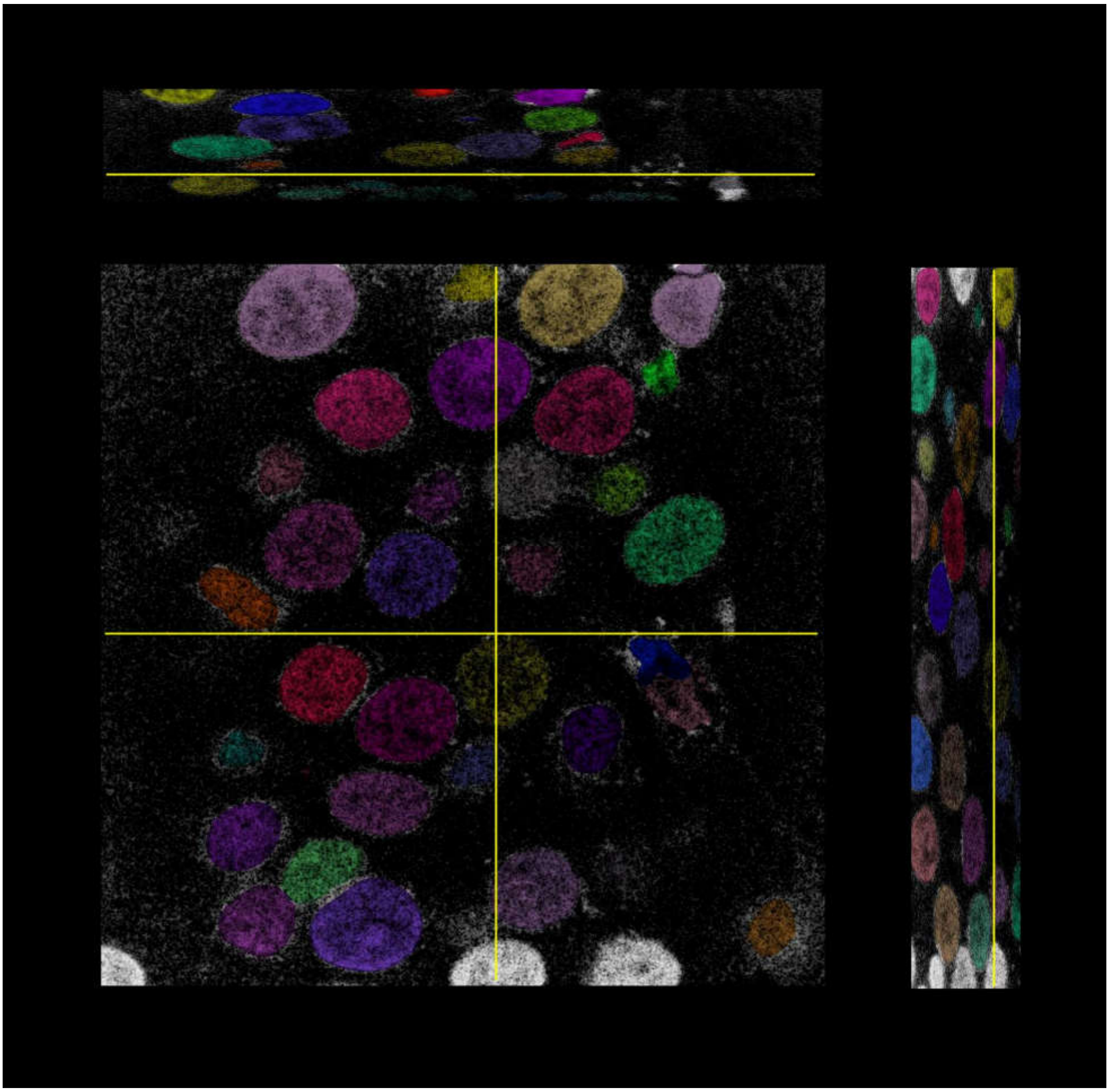

Figure S3. Segmentation results - orthogonal views

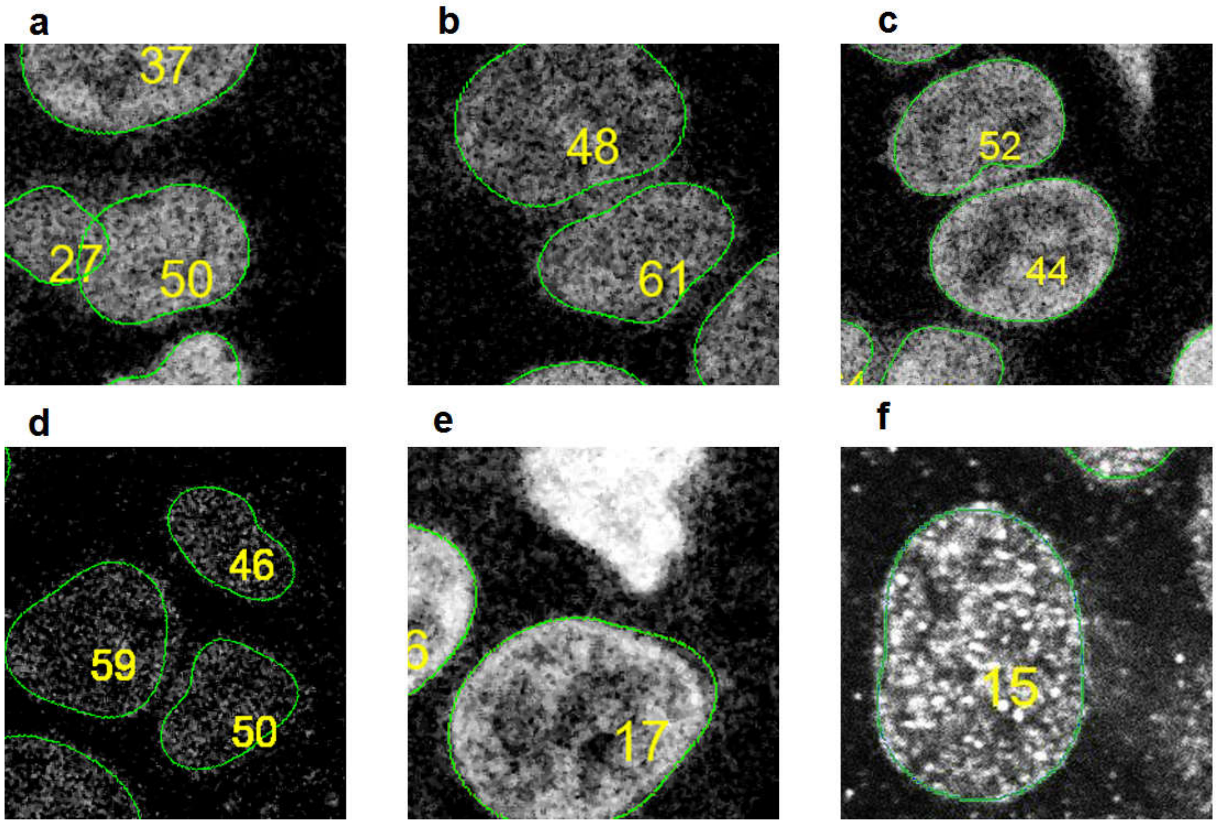

Figure S4. Examples of nuclei segmented with defects (NP category): a) part of nuclei overlaps, b) lack of clear border between nuclei, some signal does not belog to either af adjacents nuclei, c) and d) the boundary gets inside the actual nuclei, e) the boundary separated fron the actual nuclei) f) the boundary does not natch exactly the nucleus surface

S IV. Algorithm parameters

Parameter Name	Description	Typical Value
Maximal depth	Limit on maximal dimension of nucleus in z-direction	15-25 μm
Minimal depth	Minimal dimension of nucleus in z-direction	1-2 μm
Minimal diameter	The seed point is detected if the nucleus on the x-y section is larger than the circle with prescribed diameter	2-3 μm
Maximal diameter	The seed point is detected if the nucleus on the x-y section is smaller than the circle with prescribed diameter	5-8 μm
Minimal seed point separation	Assumed minimal separation between the seed points, if the algorithm detects two ore more seed points closer than the prescribed distance, the seed points with the lower priority weight are filtered out	5-8 μm
Minimal value of priority weight	seed points with the value of priority weight lower than prescribed value are filtered out.	1.2-2
Minimal value of the quality estimator Q	The minimal value of the quality estimator Q, that controls when to abort the segmentation of nucleus, and to procced with next seed point on the list	0.4-0.6
Points resolution	number of points parameterizing the boundary, on a single 2-dim section	100-500
Maximal intracluster deviation	Maximal deviation between the neighbourgh points in a single cluster	0.5-1 μm
Z-direction margin	parameter controlling how much the boundary can vary between two adjacent z plane	1-1.5 μm

Table S1. Overview of tunable algorithm parameters
